# Vaginal Microbiota Patterns Associated with Yeast Infection in Mexican Women, a Pilot Study

**DOI:** 10.3390/biotech14020031

**Published:** 2025-04-26

**Authors:** Janet Pineda-Díaz, Carolina Miranda-Brito, Carmen Josefina Juárez-Castelán, Alberto Piña-Escobedo, Noemí del Socorro Lázaro-Pérez, Alejandra de la Cruz-Munguía, Daniela Ramírez-Sánchez, Yuliana Gómez-Meraz, Juan Manuel Vélez-Ixta, Jaime García-Mena

**Affiliations:** 1 Departamento de Genética y Biología Molecular, Cinvestav, Av. Instituto Politécnico Nacional 2508, Mexico City 07360, Mexico; jpineda@cinvestav.mx (J.P.-D.); cmiranda@cinvestav.mx (C.M.-B.); carmen.juarez@cinvestav.mx (C.J.J.-C.); apinae@cinvestav.mx (A.P.-E.); noemi.lazaro@cinvestav.mx (N.d.S.L.-P.); alejandra.delacruz@cinvestav.mx (A.d.l.C.-M.); danielaramireza23@gmail.com (D.R.-S.); juan.velez@cinvestav.mx (J.M.V.-I.); 2 Ginecología y Obstetricia, Centro Médico ABC Santa Fe, Av. Carlos Graef Fernández 154, Mexico City 05300, Mexico; ygm85@hotmail.com

**Keywords:** RVVC, *Candida albicans*, *Nakaseomyses glabratus*, *Candida glabrata*, *Saccharomyces cerevisiae*, vaginal microbiota, 16S rRNA

## Abstract

Recurrent vulvovaginal candidiasis (RVVC) is a common condition that affects women of reproductive age. The etiology of RVVC remains largely unknown, but it is believed to be associated with changes in vaginal microbiota composition. This study investigates the vaginal microbiota in 57 women with RVVC and 38 healthy controls. Bacterial DNA was analyzed using high-throughput 16S rRNA gene sequencing, and *Candida* and *Saccharomyces* species were determined by PCR. RVVC cases had a higher prevalence of *Nakaseomyses glabratus* (former *Candida glabrata*) compared to controls. Alpha diversity metrics were similar between groups, but beta diversity analysis revealed significant differences in vaginal microbiota composition. The Firmicutes abundance was altered in RVVC cases, with genus *Bifidobacterium* and phylum Actinobacteriota being more abundant than in the controls. At the genus level, *Lactobacillus* dominated controls using antibiotics, while *Bifidobacterium* was higher in cases with no antibiotic intake. Our study provides evidence that *Nakaseomyses glabratus* (former *Candida glabrata*) is a significant pathogen in RVVC, while *Candida albicans* was more prevalent in healthy women. The vaginal microbiota composition differs significantly between the two groups, with distinct patterns of bacterial abundance and changes in Firmicutes abundance.

## 1. Introduction

The human body is inhabited by many microorganisms, bacteria, archaea, and fungi, collectively known as microbiota [1]. Among them, vaginal microbiota constitutes about 9% of total human microbiota and comprises a diverse array of beneficial microbes and opportunistic pathogens that inhabit the vaginal milieu [2,3]. The organisms that inhabit the vagina are dynamic and respond uniquely to various factors such as diet, exercise, medications, and diseases, which cause bacterial dysbiosis. This disruption increases the risk of contracting different urogenital microbial and fungal infections, which can be caused by sexually transmitted infections (STIs) or pelvic inflammatory disease (PID) [2,4].

Some of the characteristic symptoms of vaginal infections occurring in women of reproductive age are foul odor, burning, itching, and presence of discharge [5]. In the case of pregnant women, this microbial ecosystem changes according to the maturation stages of the woman, for example, weeks after childbirth, maternal estrogen metabolism promotes the proliferation of various aerobic and anaerobic bacteria due to mucosa thinning, an increase in vaginal pH, and a reduction in the glycogen substrate [6]. Lactobacillus of genera *L. jensenii*, *L. inerss*, *L. gasseri*, *L. crispatus*, and *Gardnerella vaginalis* mainly dominate the vaginal microbial community of healthy women in reproductive age [1,2], and in the last trimester of pregnancy [7]. These microorganisms prevent the invasion of pathogens, keeping the population in control. Previous work focusing on Mexican women using PCR and denaturing gradient gel electrophoresis (PCR-DGGE) reported bacterial communities in the vaginal tract of Mexican women during pregnancy, identifying 21 microorganism species, with 62.5% belonging to the genus *Lactobacillus*, 8.5% to non-cultivated *Ureaplasma*, 6.7% to BVAB1, and 3.0% to *Gemella bergeriae* [8].

In the case of fungi in humans, members of the fungal genera *Candida* are commonly found on the skin and mucous membranes, producing in some cases an opportunistic vaginal infection called candidiasis. *Candida albicans* is one of the leading agents associated with vulvovaginal candidiasis (VVC), a multifactorial infectious disease of the female reproductive tract that predominates during fertile years in women [9]. Some studies report that microbial diversity characterized by low *Lactobacillus* abundance is associated with low levels of inflammation, and high non-*Lactobacillus* dominated diversity (dysbiosis) is associated with increased risk of infection and obstetric complications [1,10].

Furthermore, fungi, especially yeast *Candida*, are found in vaginal mucosa as symbionts, forming a complex vaginal ecosystem with other bacteria. Fungi play diverse roles, such as lowering vaginal pH, producing bioactive compounds, competing for nutrients, adhesion sites, and regulating host immune responses [4,11]. It is reported that an overgrowth of *Candida*, specifically *C. albicans*, characterizes VVC, one of the most common vaginal infections, and inflammation. This fungus is one of the most abundant species in premenopausal women, pregnant women, and women with acute VVC [4]. The haploid fungi *Nakaseomyses glabratus* (former *Candida glabrata*) may be a regular component of the epithelial microbiota of the skin, oral cavity, gastrointestinal, and urogenital tract. Also known as an opportunistic pathogen, it is associated with different vertebrate microbiotas. This fungus is reported in Europe and North America as the second cause of candidiasis and has recently been reclassified as *Nakaseomyses glabratus* [12,13].

The purpose of this work was to characterize vaginal bacterial diversity in conditions of yeast infection in a sample of Mexican women to identify taxa signatures with a potential association with the presence of *Nakaseomyses glabratus* (former *Candida glabrata*) and or *Candida albicans.*

## 2. Materials and Methods

### 2.1. Experimental Design

This study recruited Mexican women aged 18–45 from gynecological consultations at the ABC Medical Center in Mexico City (19°23′57.4026″ N 99°11′47.57136″ W), using a convenient sampling approach. Participants were divided into two groups. Cases were women with recurrent vulvovaginal candidiasis (RVVC), having at least four episodes in the past 12 months, while controls were healthy women attending routine annual Papanicolaou test (pap smear test) consultations, without a history of recurrent vaginitis. Inclusion criteria for both groups required abstinence from sexual activity for 48 h, no menstruation at the time of sample collection, and no diagnosis of diabetes mellitus, obesity, or autoimmune diseases. Cases were additionally required to have a history of RVVC, while controls were limited to a history of one or fewer vaginitis episodes over their lifetime. Exclusion criteria included menstruation during sample collection or declining to participate. Controls were excluded if they had symptoms of vaginitis within the past six months or a history of more than one vaginitis episode diagnosed as vulvovaginal candidiasis. Cases were excluded if they did not meet the number of RVVC episodes or the specified age range. All participants provided informed consent following the 2013 Declaration of Helsinki after receiving a detailed explanation of the study. The protocol was approved by the Committee of Research and the Ethics in Research Committee of the ABC Medical Center (Project identification code: 2016-06-23). Samples were collected from June 2016 to May 2017.

### 2.2. Vaginal Swab Sampling

Participants were positioned on a gynecological examination table with stirrups, and a vaginal speculum was inserted for sample collection. Cervicovaginal mucus was collected using Catch-All™ Sample Collection Swabs (Epicentre, Illumina, San Diego, CA, USA, Cat# QEC89100) from the vaginal fornix. The vaginal pH was measured using Hydrion pH test strips (MicroEssential, Brooklyn, NewYork, NY, USA, Cat#165/1-12). The swab trimmed to fit was then placed in a 2 mL tube containing sterile PBS solution pH 7.0 and stored at 4 °C until DNA extraction.

### 2.3. DNA Extraction from Vaginal Swab Samples

DNA was extracted from vaginal swab samples using the GeneAll Exgene Stool SV Kit (Gene All, Songpa-gu, Seoul, Korea, Cat#115-150). The samples were centrifuged for 5 min at 3800× *g*, and the supernatant was discarded. The pellet was resuspended in 1.0 mL of PBS buffer pH 7.0, vortexed for 1 min, and incubated at room temperature for 30 s. After centrifugation at maximum speed for 2 min, the supernatant was discarded. The pellet was resuspended in 1.3 mL of Buffer FL, incubated for 5 min at room temperature, and centrifuged at ≥10,000× *g* for 5 min. The supernatant was transferred to an EzPass filter column, centrifuged, and eluted into a clean 1.5 mL tube with 100 µL of Buffer EB. After further purification steps involving Buffer PB and Buffer NW, the final DNA elution was performed with 50 µL of Buffer EB. DNA integrity and concentration were verified using a Nanodrop 2000 spectrophotometer (Thermo Scientific; Waltham, MA, USA, Cat#ND-2000) and 0.5% agarose gel electrophoresis.

### 2.4. 16S rRNA Gene Library Preparation

The V3 polymorphic region of bacterial 16S rRNA gene, ~281 bp in length, was amplified using primer V3-341F series (positions 340–356 of *Escherichia coli* 16S rDNA molecule *rrnB* GenBank accession number J01859.1), containing different 12 pb Golay barcode, and antisense primer V3-518R (complementary to positions 517–533) (Table S1). The PCR was carried out in the GeneAmp PCR System 2700 Thermal Cycler (Applied Biosystems, ThermoFisher Scientific, Waltham, MA, USA), and the program was 5 min 95 °C; 25 cycles [15 s, 94 °C; 15 s, 62 °C; 15 s, 72 °C] followed by an extension of 10 min at 72 °C [14].

### 2.5. High-Throughput DNA Semiconductor Sequencing

Equal concentrations of amplicons were pooled and purified, subsequently sequenced as previously reported [14]. DNA library concentration and final fragment size were measured with the 2100 Bioanalyzer Instrument fragment analyzer (Agilent Technologies, Santa Clara, CA, USA), and the average library size obtained was 263 bp. Emulsion PCR was carried out using Ion OneTouchTM 200 Template Kit v2 DL (Life Technologies, Carlsbad, CA, USA). Amplicon enrichment with ionic beads was carried out using Ion OneTouch ES (Life Technologies, Carlsbad, CA, USA). The Ion 318 Kit V2 chip (Cat. 4488146, Life Technologies, Carlsbad, CA, USA) and the Ion Torrent PGM system were used for sequencing. The reads were filtered using PGM software to eliminate polyclonal (homopolymers > 6) and low-quality sequences (quality score ≤ 20).

### 2.6. Yeast Identification by PCR Targeting ITS1 and ITS2 and MEX67 Gene

To characterize the etiology of RVVC in samples from the participants, PCR reactions were performed using species-specific primers for *C. albicans* and *N. glabratus* (former *C. glabrata*), targeting the ITS1 and ITS2 of *C. albicans* and *C. glabrata*, respectively (Table S2) [13,15]. Primers for *S. cerevisiae* targeting the nuclear export MEX67 gene [16] (Table S2) were included due to the potential involvement of this yeast in vulvovaginitis, which clinically resembles *Candida* infections. For ITS, the PCR program followed the initial denaturation step at 96 °C for 5 min, followed by 30 cycles [30 s, 94 °C, 30 s, 58 °C, and 30 s, 72 °C], a final extension at 72 °C for 15 min, cooling to 10 °C for 10 min; for MEX67 initial denaturation step at 94 °C for 5 min, followed by 30 cycles [60 s, 94 °C, 60 s, 58 °C, and 60 s, 72 °C], and a final extension at 72 °C for 5 min, cooling to 10 °C for 5 min. Positive controls included *C. albicans* ATCC, *C. glabrata* CBS138, and *S. cerevisiae* S2886, and negative controls used reaction mixtures without DNA templates. Amplicons of ~273 bp, 423 bp, and 150 bp were fractionated in 2.0% agarose gel electrophoresis stained with Midori Green Advanced dye(Nippon Genetics^®^, Dueren, Germany), and products were visualized using the Molecular Imager Gel Doc XR system Mod. Universal Hood II (Bio-Rad, Hercules, CA, USA, Cat#8195). The three PCR products were cloned using the GeneJET PCR Cloning Kit (ThermoFisher Scientific, Waltham, MA, USA), and plasmid DNA was extracted for Sanger capillary sequencing with pJET1 primers [15]. Resulting sequences were analyzed using VECTOR NTI Advance and BLAST alignments to verify species-specific diagnostic accuracy.

### 2.7. ASV Determination and Taxonomic Annotation

The FASTQ files were further processed and analyzed using QIIME 2022.2 [17]. Amplicon Sequence Variants (ASVs) were determined with the QIIME dada2 denoise-single plugin, with sequence truncation at 238 nt. Taxonomic assignment was performed using the feature-classifier classify-consensus-blast plugin with a 99% percentage identity. The Greengenes 2 database was used for BLAST analysis (QIIME 2022.2) [18].

### 2.8. Bioinformatic Analyses

The analyses were conducted with RStudio in R 4.2.0 [19,20]. The following packages were employed for relative abundance, diversity and differential analyses: phyloseq 1.4.0 [21], DESeq2 1.3.6 [22], and ALDEx2 1.36.0; qiime2R [23] to import the qiime artifacts [24]; for alpha and beta diversity analyses vegan 2.6-2 [25]; lefser 1.15.9 [26] for discriminatory analyses; ComplexHeatmap 2.12.0 [27] for heatmap elaboration; and tidyverse 1.3.1 [28], dplyr 1.09, ggplot2 3.3.6, scales 1.2.0, ggpubr 0.4.4, and gridExtra 2.3 [29] for graphical images.

## 3. Results

### 3.1. Characteristics of Participants and Candida Prevalence in Vaginosis Cases vs. Controls

**Figure 1:**
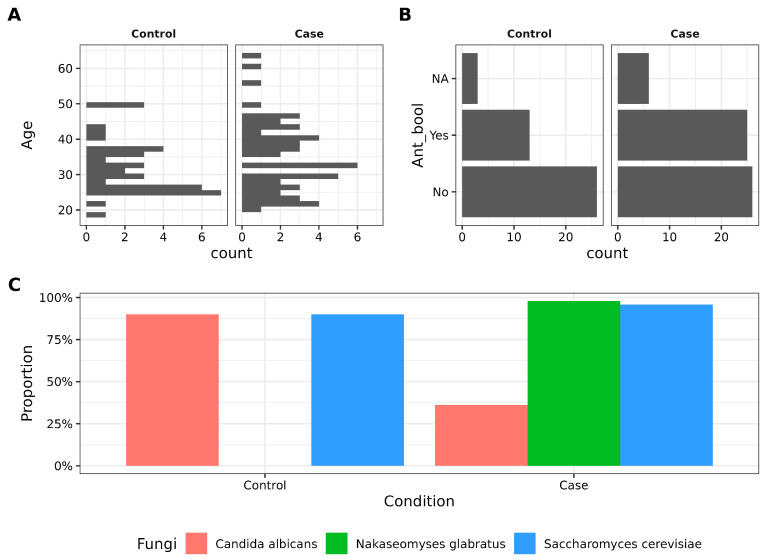
Main variables driving the study. (**A**). Histogram depicting the age (in years) distribution between control and case groups. No statistical difference was found according to the Student’s *t*-test. (**B**). Barplot showing antibiotic intake in women. (**C**). Barplot showing the proportion of control and case groups with three different fungi detected by PCR.

In this study, 38 controls and 57 cases met the inclusion criteria, of which 27 controls and 54 cases were successfully sequenced (Table S3). No statistically significant differences were observed between groups regarding age, age of menarche, sexual life start, or vaginal pH, and other variables showed similar distribution between groups (Figure S1). A subset of participants, 13 cases (30.95%), and 25 controls (43.86%) reported the use of antibiotics in the last three months, and this factor was considered in subsequent analyses. Additionally, PCR detection revealed significant differences in the prevalence of *C. albicans* (90% in controls vs. 36.17% in cases) and *N. glabratus* (0.0% controls vs. 97.87% cases) based on two-proportion z-tests (Figure 1C, Table 1). On the other hand, *S. cerevisiae* showed a comparable abundance in both groups (90.0% controls vs. 95.74% cases).

### 3.2. Antibiotic Intake and Patient Condition Have a Small Effect on Beta Diversity

**Figure 2:**
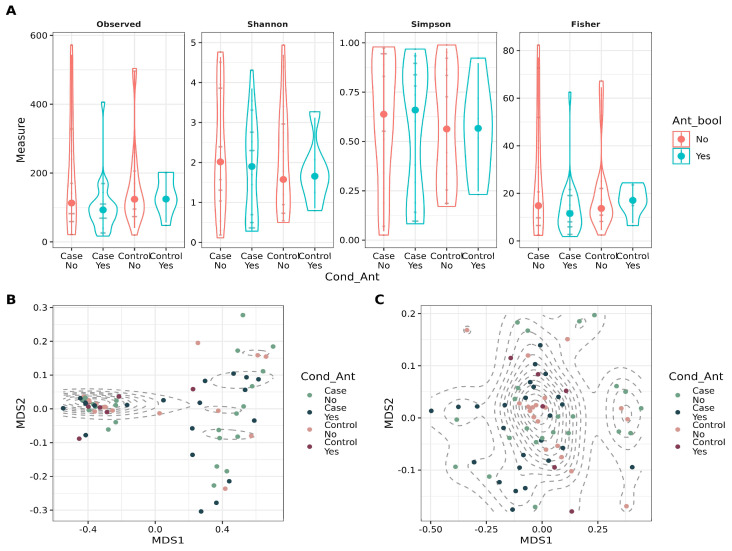
Diversity metrics for stratified groups, considering antibiotic intake and condition (case vs. control). (**A**). Violin plot depicting alpha diversity metrics (Observed number of ASVs, Shannon, Simpson, and Fisher) for the groups. (**B**). Scatter plot showing Non-Metric Multidimensional Scaling (NMDS) for weighted Unifrac distance for beta diversity assessment in the groups. ADONIS was applied to account for the variance explained by Condition (*p* = 0.036, $R^{2}$ = 0.05) and Antibiotic intake (*p* = 0.39, $R^{2}$ = 0.01). (**C**). Scatter plot showing Non-Metric Multidimensional Scaling (NMDS) for unweighted Unifrac distance for beta diversity assessment in the groups. ADONIS was applied to account for the variance explained by Condition (*p* = 0.012, $R^{2}$ = 0.03) and Antibiotic intake (*p* = 0.018, $R^{2}$ = 0.03).

The sequencing data were processed as described in the Section 2. For samples with low sequencing depth, re-sequencing was performed, and the resulting reads were combined by summation (Figure S2). Both alpha and beta diversity metrics were analyzed to investigate the impact of antibiotic use and patient condition on global bacterial diversity. While alpha diversity appeared unaffected by these factors, beta diversity showed significant associations based on ADONIS tests. Weighted beta diversity was influenced primarily by patient condition (*p* = 0.042), whereas unweighted beta diversity was significantly associated with both patient condition (*p* = 0.012) and antibiotic use (*p* = 0.018). Despite these findings, the low $R^{2}$ values indicate a small effect (Figure 2). Additionally, sequencing depth was examined with antibiotic consumption to determine whether it contributed to reduced read counts, yet no evidence of such an effect was found (Figure S3).

### 3.3. Vaginal Microbiota Composition Reflects RVVC Condition and Antibiotic Use

**Figure 3:**
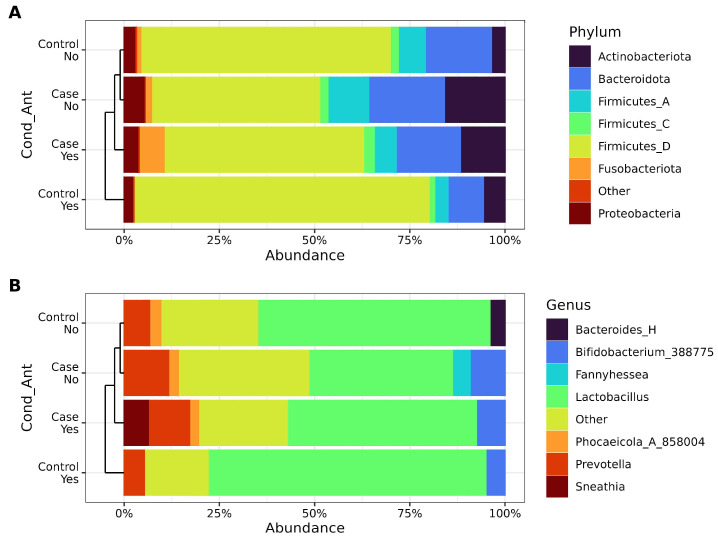
Relative abundance for stratified groups, considering antibiotic intake and condition (case vs. control). (**A**). Phylum relative abundance as percentages. (**B**). Genus relative abundance as percentages. Taxa are identified on the right side of each figure.

The composition of the vaginal microbiota was analyzed at both the phylum (Figure 3A) and genus levels (Figure 3B) to identify changes in key bacterial taxa across samples. Hierarchical clustering using weighted beta diversity was employed to compare taxa and assess similarities among bacterial communities in the groups. Samples were first grouped by antibiotic intake and subsequently by RVVC condition. Significant alterations were observed in the Firmicutes abundance, particularly between cases and controls. Cases without antibiotic use exhibited the highest abundance of Bacteroidota and Actinobacteriota phyla, whereas controls who had taken antibiotics were dominated by the Firmicutes_D phylum.

At the genus level, the members of the phylum Firmicutes_D were predominantly represented by *Lactobacillus*, which also dominated controls with antibiotic use. In contrast, cases without antibiotic intake showed a higher prevalence of *Bifidobacterium*, while *Bacteroides_H* was found only in controls who had not taken antibiotics. Notably, *Fannyhessea* was characteristic of cases without antibiotic use, whereas *Sneathia* was specific to cases with antibiotic use. Additionally, a marked increase in genus *Prevotella* was observed in cases where *Lactobacillus* was replaced by a diverse array of low-abundance taxa (<2%, grouped as “Other”). These findings highlight the dynamic shifts in microbial composition associated with antibiotic use and patient condition (Figure 3B).

### 3.4. Lactobacillus and Other Key Bacteria Are Associated with Healthy Vaginal Microbiota

**Figure 4:**
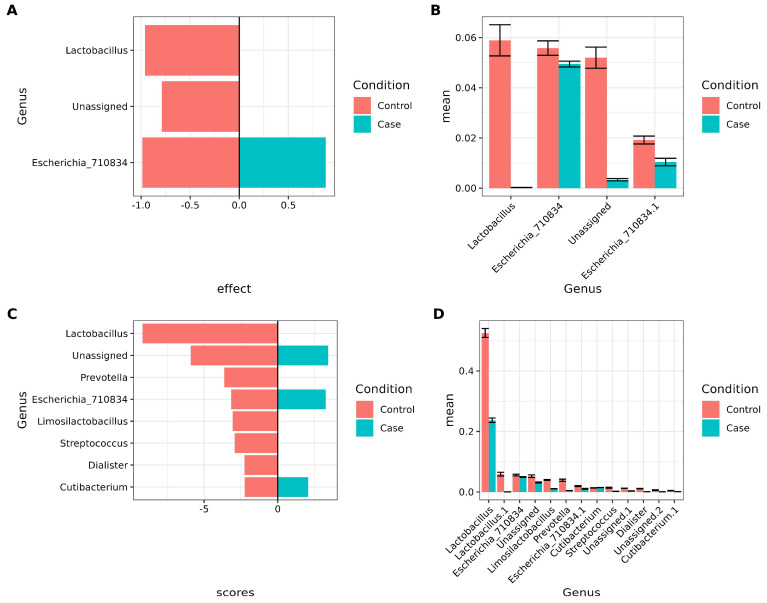
Important biomarker bacteria considering antibiotic intake and condition. (**A**,**B**), Using ALDEx2 (“y∼ antibiotic intake + RVVC condition”). (**C**,**D**), using LEFSE (Class = RVVC, Subclass = antibiotic intake). Only features with p-values that passed FDR correction were included in the graphs. (**A**). Barplot showing ALDEx2 results for condition (case vs. control). The X-axis shows the effect, and the Y-axis shows the genus. Negative values correspond to control, positive values correspond to cases. (**B**). Barplot of mean sqrt of relative abundance of taxa found by ALDEx2. The X-axis shows the mean sqrt of relative abundance, and the Y-axis shows the genera. (**C**). Barplot showing LEFSE results for condition (case vs. control). The X-axis shows the LDA scores, and the Y-axis shows the genus. Negative values correspond to control; positive values correspond to cases. (**D**). Barplot of mean sqrt of relative abundance of taxa found by LEFSE. The X-axis shows the mean sqrt of relative abundance, and the Y-axis shows genus.

Differential abundance analyses were performed to identify bacteria associated with vaginosis while accounting for antibiotic intake among participants. Two approaches were used: ALDEx2, with the formula “y∼ antibiotic intake + RVVC condition”, and linear discriminant analysis (LEfSe), using RVVC as the class variable and antibiotic intake as the subclass. The results from ALDEx2 are presented in Figure 4A, with corresponding square root-transformed relative abundance values shown in Figure 4B. In the control group, *Lactobacillus* and an unassigned taxon were prominent. Interestingly, two distinct ASVs classified as *Escherichia*_710834 were identified in both the control and case groups.

LEfSe results are shown in Figure 4C, with the corresponding square root-transformed relative abundance values in Figure 4D. These findings corroborate the ALDEx2 results for *Lactobacillus*. In addition, *Prevotella*, *Limosilactobacillus*, *Streptococcus*, and *Dialister* were predominant in controls, whereas two distinct *Escherichia*_710834 ASVs, unassigned taxa, and *Cutibacterium* were characteristic of cases.

## 4. Discussion

This work aims to characterize the vaginal microbiota of women suffering from RVVC. We found no differences between cases and control in women associated with metadata. For instance, no differences in vaginal pH were consistent with the disease diagnosis [30]. The age prevalence in women is also consistent with the literature, which indicates that most episodes occur in the range from 19 to 35 years with lower prevalence rates after 50 years [31]. In our sample, we observed a broader range between 20 to 50 years, with most cases around 35. Additionally, an association between RVVC incidence and new sexual partners is reported [32]. In our work, there is a tendency among cases to have more sexual partners, but our results are not conclusive in this matter.

In this work, we detected by PCR *N. glabratus* (former *C. glabrata*) exclusive to RVVC cases, while *C. albicans* were found in higher proportion in the control group. Interestingly, some reports mention *C. albicans* as the major cause of infection, with 80–95% of isolates identified as this species [30]. Some other species, such as *N. glabratus* (formerly *C. glabrata*), *Candida tropicalis*, and *Candida parapsilosis*, are now frequently identified as human pathogens [33]. Moreover, *Candida* spp. has been previously isolated from healthy women [34,35], and it has been reported that *Candida* infections are common and, in most cases, occur in non-symptomatic healthy individuals [36]. Due to the contrasting results in this study, there might be an effect of the vaginal bacteria in our participants, which will be Fexplored further. It was also interesting, for the scope of this study, that a significant portion of participants in both groups reported the use of antibiotics. It has been reported that the intake of broad-spectrum antibiotics might reduce gut microbiota diversity [37]. For this reason, we had to take antibiotic intake into account when evaluating the bacterial profile.

We found no major differences in alpha diversity between cases and controls in our work. However, a small effect was observed due to both antibiotic intake and RVVC. As mentioned before, it is expected that antibiotics have an impact on microbiota diversity, also in the context of vaginal microbiota, and in the long term might develop resistance and cause recurrent infections [38]. Overall, the diversity in all groups was low, which is expected in the case of vaginal microbiota [4], for instance, *Clostridioides difficile* infection in the gut could occur due to dysbiosis caused by antibiotics [39]. Previous reports evaluating microbial diversity during RVVC conditions found major differences in alpha and beta diversity, with lower diversity in the cases [40,41].

Our relative abundance results indicate an increase in *Bifidobacterium* in association with RVVC, while antibiotic intake exhibited a mixed effect, increasing *Bifidobacterium* in controls and decreasing in the cases. In a previous study, a reduction in gut *Bifidobacterium* was observed in response to amoxicillin and azithromycin [42]. *Gardnerella vaginalis* has been widely associated with vaginal infections [43,44], and recently, ATCC Genome Portal reclassified *Gardnerella vaginalis* as part of *Bifidobacterium* [45]. In two previous works, studying women with bacterial vaginosis exposed to metronidazole or clindamycin, mixed effects in *Gardnerella* abundance were observed after the treatment, increasing in some women and decreasing in others [46,47]. Furthermore, we found *Sneathia* in cases where the participant consumed oral antibiotics. Previous work showed *Sneathia* was unchanged by antibiotic intake [47]. There is sufficient evidence pointing to *Sneathia* as an emerging pathogen in female reproductive disease with adverse perinatal outcomes [48].

Additionally, we identified *Lactobacillus* with ALDEx2 and LEFSE as characteristic of healthy women when comparing to RVVC. This is consistent with other works [2,49], which have linked *Lactobacillus* to a healthy vagina when studying bacterial vaginosis. Remarkably, we found different ASVs classified as *Escherichia* in our study, characterizing both cases and controls. Another report found *Escherichia* to be predominant in patients with aerobic vaginitis [49,50]. Particularly, other bacteria identified by LEFSE analysis and associated with healthy women were *Prevotella*, *Limosilactobacillus*, *Streptococcus*, and *Dialister*. The association between Bacterial Vaginosis and increased abundance of anaerobes like *Prevotella*, which is an important source of lipopolysaccharide and ammonia in vaginal mucus, enhances the growth of other bacterial vaginosis-associated organisms like *Gardnerella vaginalis* [50,51]. In the case of probiotics, *Limosilactobacillus*, one ex-member of the *Lactobacillus* species, is a probiotic bacterium that can be found in various parts of the human body, like the gastrointestinal tract (GI), urinary tract, skin, and breast milk, and suggests its metabolites promote human health [51,52]. In the case of *Streptococcus*, it has been associated with Bacterial Vaginosis in pregnant women, which also presents a dysbiosis of *Lactobacillus* [53]. The genus *Dialister* also has been associated with higher prevalence and abundance in Bacterial vaginosis [41].

## 5. Conclusions

We observed some significant differences in vaginal microbiota composition between women with recurrent vulvovaginal candidiasis (RVVC) and healthy controls. *N. glabratus* (formerly *C. glabrata*) was exclusively detected in RVVC cases, while *C. albicans* was more prevalent in the control group. The vaginal microbiota diversity was relatively low, but we found distinct patterns of bacterial abundance between the two groups. *Bifidobacterium* exhibited an increase in response to RVVC, while antibiotic intake had a mixed effect on bacterial abundance, decreasing it in cases and increasing it in controls. The pathogen *G. vaginalis* (classified as part of *Bifidobacterium*) showed variable responses after antibiotic treatment in both groups of this study. *Lactobacillus* was more prevalent in healthy women when contrasted to RVVC. Other bacteria associated with healthy women included *Prevotella*, *Limosilactobacillus*, *Streptococcus*, and *Dialister*. These observations underscore the importance of further research into the mechanisms behind these associations, with a particular focus on the interactions between *N. glabratus* and vaginal bacteria.

## Figures and Tables

**Table 1:** General data for participants of the study.

Variable	Control	Cases	*p*-Value
Number	38	57	-
Age	31.92 (±7.67) (38)	35.31 (±10.22) (51)	0.08
Menarche Age	12.53 (±1.59) (38)	12.64 (±1.44) (51)	0.71
Age Start Sexual Life	19.67 (±3.00) (36)	18.62 (±2.89) (48)	0.11
Contraceptive Method			
None	14/32 (43.75%)	29/57 (50.88%)	-
Tubal ligation	1/32 (3.12%)	8/57 (14.03%)	-
Condom	6/32 (18.75%)	10/57 (17.54%)	-
Intrauterine device	2/32 (6.25%)	6/57 (10.53%)	-
Oral	7/32 (21.88%)	4/57 (7.02%)	-
Implant	2/32 (6.25%)	0/57 (0.00%)	-
Menstrual Cycle Phase			
Luteal	19/32 (59.38%)	32/55 (58.18%)	-
Follicular	11/32 (34.38%)	17/55 (30.91%)	-
Ovulation	2/32 (6.25%)	6/55 (10.91%)	-
Sex Partners			
1	15/32 (46.88%)	21/54 (38.89%)	-
≥2	17/32 (53.13%)	33/54 (61.11%)	-
Gestations			
0	17/31 (54.84%)	17/52 (32.69%)	-
1–2	7/31 (22.58%)	18/52 (34.62%)	-
≥3	7/31 (22.58%)	17/52 (32.69%)	-
Delivers			
0	22/31 (70.97%)	32/52 (61.54%)	-
1–2	4/31 (12.90%)	13/52 (25.00%)	-
≥3	5/31 (16.13%)	7/52 (13.46%)	-
Cesarean sections			
0	25/31 (80.65%)	30/52 (57.69%)	-
1–2	5/31 (16.13%)	21/52 (40.38%)	-
≥3	1/31 (3.23%)	1/52 (1.92%)	-
Abortions			
0	29/31 (93.54%)	39/52 (75.00%)	-
1–2	1/31 (3.22%)	12/52 (23.07%)	-
≥3	1/31 (3.22%)	1/52 (1.92%)	-
Vaginal pH			
mean pH	4.48 ± 0.57 (29)	4.78 ± 1.25 (43)	0.18
≤4	14/25 (56.00%)	17/40 (42.50%)	-
≥5	11/25 (44.00%)	23/40 (57.50%)	-
Risk factors			
Smoking	8/42 (19.05%)	19/58 (33.33%)	-
Medication			
Antibiotics	13/42 (30.95%)	25/57 (43.86%)	-
Antifungal	0/33 (0.00%)	5/57 (8.77%)	-
Vulvovaginal Candidiasis episodes			
0	31/31 (100.00%)	0/52 (0.00%)	-
1–2	0/31 (0.00%)	38/52 (73.08%)	-
≥3	0/31 (0.00%)	14/52 (26.92%)	-
PCR *Candida* detection			
*Candida albicans*	27/30 (90.00%)	17/47 (36.17%)	9.93 × 10^−6^
Nakaseomyses glabratus *	0/30 (0.00%)	46/47 (97.87%)	<2.2 × 10^−16^
*Saccharomyces cerevisiae*	27/30 (90.00%)	45/47 (95.74%)	0.6000

The mean is accompanied by (±), representing the standard deviation. ( ) is the number of participants with available data. Categorical data are shown as proportions; percentages are enclosed in parentheses. For age, menarche age, the start of sexual life, and pH, a Student’s *t*-test was applied. A two-proportion z-test was used for the PCR detection results. * Former *Candida glabrata*.

## Data Availability

The datasets generated for this study can be found in the NCBI BioProject ID PRJNA1199545 Link https://www.ncbi.nlm.nih.gov/bioproject/PRJNA1199545 (accessed on 10 April 2025).

## Supplementary Materials

The following supporting information can be downloaded at: https://www.mdpi.com/article/10.3390/biotech14020031/s1, Figure S1: Typical Q-Q plot showing normal distribution of age in the studied women; Figure S2: Bar plot showing sequencing depth for each sample, and Figure S3: Bar plot showing sample counts and antibiotic intake in both case and control groups. Table S1: 5′ to 3′ sequence of barcoded primers for bacteria used in this study; Table S2: Diagnostic primers for yeast detection, and Table S3: Sequencing summary.
