# Supplementary material for: Vaginal Microbiota Patterns Associated with Yeast Infection in Mexican Women, a Pilot Study
Source: BioTech (Basel). 2025 Apr 26;14(2):31. doi: 10.3390/biotech14020031 (PMC12101427; doi:10.3390/biotech14020031)
Supplement: Supplementary file 1 [file biotech-14-00031-s001.zip › biotech-3611659-supplementary.pdf]

BioTech

MDPI

Supplementary Material

Vaginal Microbiota Patterns Associated with Yeast Infection in Mexican Women, a Pilot Study

Janet Pineda-Díaz [1, †,§], Carolina Miranda-Brito [1, †], Carmen Josefina Juárez-Castelán [1], Alberto Piña-Escobedo [1], Noemí del Socorro Lázaro-Pérez [1], Alejandra de la Cruz-Munguía [1], Daniela Ramírez-Sánchez [1, ‡], Yuliana Gómez-Meraz [2], Juan Manuel Vélez-Ixta [1,‖] and Jaime García-Mena [1,*]

1 Departamento de Genética y Biología Molecular, Cinvestav, Av. Instituto Politécnico Nacional 2508, Mexico City 07360, Mexico; jpineda@cinvestav.mx (J.P.-D.); cmiranda@cinvestav.mx (C.M.-B.); carmen.juarez@cinvestav.mx (C.J.J.-C.); apinae@cinvestav.mx (A.P.-E.); noemi.lazaro@cinvestav.mx (N.d.S.L.-P.); alejandra.delacruz@cinvestav.mx (A.d.l.C.-M.); danielaramireza23@gmail.com (D.R.-S.); juan.velez@cinvestav.mx (J.M.V.-I.)
2 Ginecología y Obstetricia, Centro Médico ABC Santa Fe, Av. Carlos Graef Fernández 154, Mexico City 05300, Mexico; ygm85@hotmail.com
* Correspondence: jgmena@cinvestav.mx
† These authors contributed equally to this work.
‡ Current address: Department of Molecular Biology, Max Planck Institute for Biology Tübingen. Max-Planck-Ring 5, 72076 Tübingen, Germany.
§ Current address: Departamento de Patología Quirúrgica y Molecular, Centro Médico ABC Observatorio, Mexico City 01120, Mexico.
‖ Department of Pediatrics, Cumming School of Medicine, University of Calgary, 2500 University Drive NW, Calgary Alberta T2N 1N4, Canada.

Figures

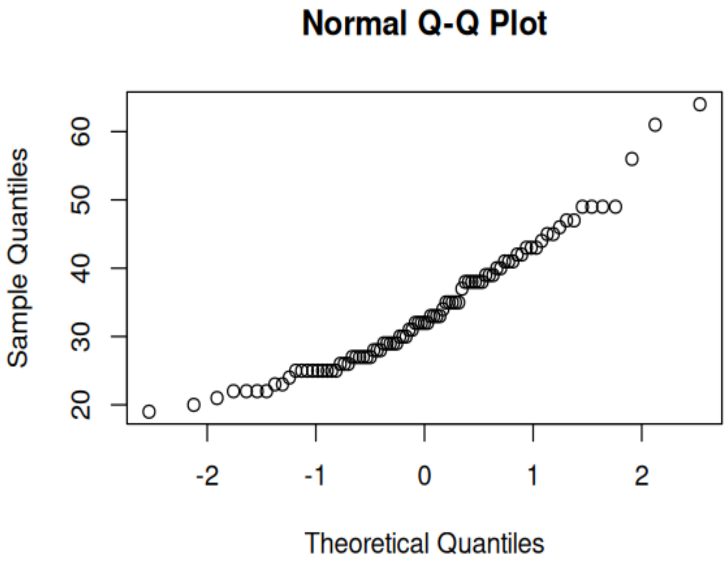

Figure S1. Typical Q-Q plot showing normal distribution of age in the studied women. Age was normally distributed according to the Shapiro-Wilk test (p-value < 0.05).

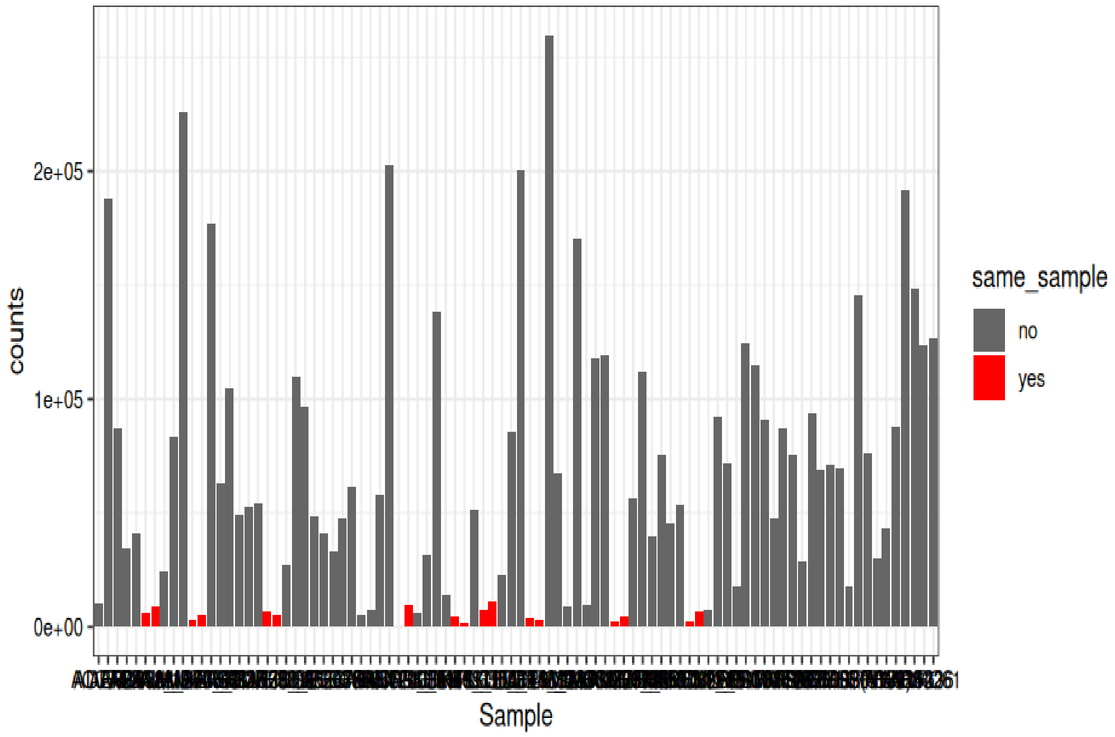

Figure S2. Bar plot showing sequencing depth for each sample. Samples highlighted in red were sequenced twice and merged because they exhibited low sequencing depth.

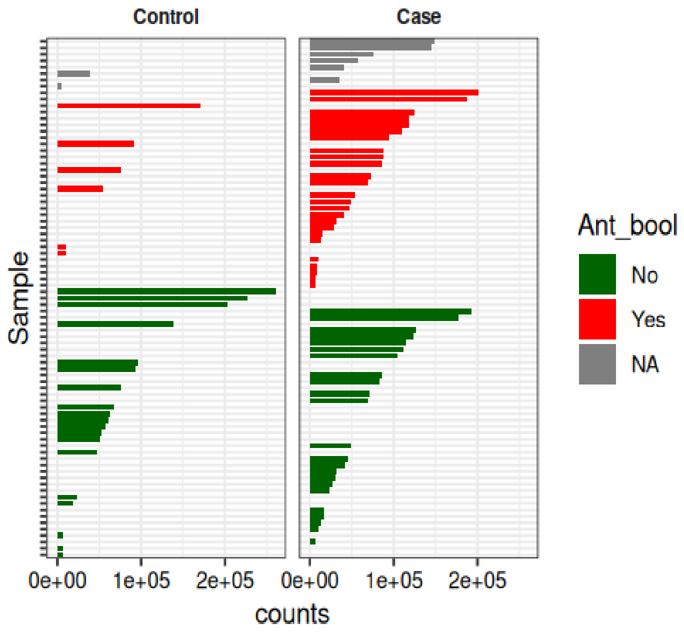

Figure S3. Bar plot showing sample counts and antibiotic intake in both case and control groups.

Tables

Table S1. 5’ to 3’ sequence of barcoded primers for bacteria used in this study.

Primer	Ion Torrent Linker	Golay Barcode	Spacer	16S rRNA gene
V3-341 F1	CCATCTCATCCCTGCGTGTCTCCGACTCAG	GATCTGCGATCC	GT	CCTACGGGAGGCAGCAG
V3-341 F2	CCATCTCATCCCTGCGTGTCTCCGACTCAG	CAGCTCATCAGC	GT	CCTACGGGAGGCAGCAG
V3-341 F3	CCATCTCATCCCTGCGTGTCTCCGACTCAG	CAAACAACAGCT	GT	CCTACGGGAGGCAGCAG
V3-341 F4	CCATCTCATCCCTGCGTGTCTCCGACTCAG	GCAACACCATCC	GT	CCTACGGGAGGCAGCAG
V3-341 F5	CCATCTCATCCCTGCGTGTCTCCGACTCAG	GCGATATATCGC	GT	CCTACGGGAGGCAGCAG
V3-341 F6	CCATCTCATCCCTGCGTGTCTCCGACTCAG	CGAGCAATCCTA	GT	CCTACGGGAGGCAGCAG
V3-341 F7	CCATCTCATCCCTGCGTGTCTCCGACTCAG	AGTCGTGCACAT	GT	CCTACGGGAGGCAGCAG
V3-341 F8	CCATCTCATCCCTGCGTGTCTCCGACTCAG	GTATCTGCGCGT	GT	CCTACGGGAGGCAGCAG
V3-341 F9	CCATCTCATCCCTGCGTGTCTCCGACTCAG	CGAGGGAAAGTC	GT	CCTACGGGAGGCAGCAG
V3-341 F10	CCATCTCATCCCTGCGTGTCTCCGACTCAG	CAAATTCGGGAT	GT	CCTACGGGAGGCAGCAG
V3-341 F11	CCATCTCATCCCTGCGTGTCTCCGACTCAG	AGATTGACCAAC	GT	CCTACGGGAGGCAGCAG
V3-341 F12	CCATCTCATCCCTGCGTGTCTCCGACTCAG	AGTTACGAGCTA	GT	CCTACGGGAGGCAGCAG
V3-341 F13	CCATCTCATCCCTGCGTGTCTCCGACTCAG	GCATATGCACTG	GT	CCTACGGGAGGCAGCAG
V3-341 F14	CCATCTCATCCCTGCGTGTCTCCGACTCAG	CAACTCCCGTGA	GT	CCTACGGGAGGCAGCAG
V3-341 F15	CCATCTCATCCCTGCGTGTCTCCGACTCAG	TTGCGTTAGCAG	GT	CCTACGGGAGGCAGCAG
V3-341 F16	CCATCTCATCCCTGCGTGTCTCCGACTCAG	TACGAGCCCTAA	GT	CCTACGGGAGGCAGCAG
V3-341 F17	CCATCTCATCCCTGCGTGTCTCCGACTCAG	CACTACGCTAGA	GT	CCTACGGGAGGCAGCAG
V3-341 F18	CCATCTCATCCCTGCGTGTCTCCGACTCAG	TGCAGTCCTCGA	GT	CCTACGGGAGGCAGCAG
V3-341 F19	CCATCTCATCCCTGCGTGTCTCCGACTCAG	ACCATAGCTCCG	GT	CCTACGGGAGGCAGCAG
V3-341 F20	CCATCTCATCCCTGCGTGTCTCCGACTCAG	TGGACATCTCTT	GT	CCTACGGGAGGCAGCAG
V3-341 F21	CCATCTCATCCCTGCGTGTCTCCGACTCAG	GAACACTTTGGA	GT	CCTACGGGAGGCAGCAG
V3-341 F22	CCATCTCATCCCTGCGTGTCTCCGACTCAG	GAGCCATCTGTA	GT	CCTACGGGAGGCAGCAG
V3-341 F23	CCATCTCATCCCTGCGTGTCTCCGACTCAG	TTGGGTACACGT	GT	CCTACGGGAGGCAGCAG
V3-341 F24	CCATCTCATCCCTGCGTGTCTCCGACTCAG	AAGGCGCTCCTT	GT	CCTACGGGAGGCAGCAG
V3-341 F25	CCATCTCATCCCTGCGTGTCTCCGACTCAG	TAATACGGATCG	GT	CCTACGGGAGGCAGCAG
V3-341 F26	CCATCTCATCCCTGCGTGTCTCCGACTCAG	TCGGAATTAGAC	GT	CCTACGGGAGGCAGCAG
V3-341 F27	CCATCTCATCCCTGCGTGTCTCCGACTCAG	TGTGAATTCGGA	GT	CCTACGGGAGGCAGCAG
V3-341 F28	CCATCTCATCCCTGCGTGTCTCCGACTCAG	CATTCGTGGCGT	GT	CCTACGGGAGGCAGCAG
V3-341 F29	CCATCTCATCCCTGCGTGTCTCCGACTCAG	AACGCACGCTAG	GT	CCTACGGGAGGCAGCAG
V3-341 F30	CCATCTCATCCCTGCGTGTCTCCGACTCAG	ACACTGTTCATG	GT	CCTACGGGAGGCAGCAG
V3-341 F31	CCATCTCATCCCTGCGTGTCTCCGACTCAG	ACCAGACGATGC	GT	CCTACGGGAGGCAGCAG
V3-341 F32	CCATCTCATCCCTGCGTGTCTCCGACTCAG	ACGCTCATGGAT	GT	CCTACGGGAGGCAGCAG
V3-341 F33	CCATCTCATCCCTGCGTGTCTCCGACTCAG	ACTCACGGTATG	GT	CCTACGGGAGGCAGCAG
V3-341 F34	CCATCTCATCCCTGCGTGTCTCCGACTCAG	AGACCGTCAGAC	GT	CCTACGGGAGGCAGCAG
V3-341 F35	CCATCTCATCCCTGCGTGTCTCCGACTCAG	AGCACGAGCCTA	GT	CCTACGGGAGGCAGCAG
V3-341 F36	CCATCTCATCCCTGCGTGTCTCCGACTCAG	ACAGACCACTCA	GT	CCTACGGGAGGCAGCAG
V3-341 F37	CCATCTCATCCCTGCGTGTCTCCGACTCAG	ACCAGCGACTAG	GT	CCTACGGGAGGCAGCAG
V3-341 F38	CCATCTCATCCCTGCGTGTCTCCGACTCAG	ACGGATCGTCAG	GT	CCTACGGGAGGCAGCAG
V3-341 F39	CCATCTCATCCCTGCGTGTCTCCGACTCAG	AGCTTGACAGCT	GT	CCTACGGGAGGCAGCAG
V3-341 F40	CCATCTCATCCCTGCGTGTCTCCGACTCAG	AACTGTGCGTAC	GT	CCTACGGGAGGCAGCAG
V3-341 F41	CCATCTCATCCCTGCGTGTCTCCGACTCAG	ACCGCAGAGTCA	GT	CCTACGGGAGGCAGCAG
V3-341 F42	CCATCTCATCCCTGCGTGTCTCCGACTCAG	ACGGTGAGTGTC	GT	CCTACGGGAGGCAGCAG
V3-341 F43	CCATCTCATCCCTGCGTGTCTCCGACTCAG	ACTCGATTCGAT	GT	CCTACGGGAGGCAGCAG
V3-341 F44	CCATCTCATCCCTGCGTGTCTCCGACTCAG	AGACTGCGTACT	GT	CCTACGGGAGGCAGCAG
V3-341 F45	CCATCTCATCCCTGCGTGTCTCCGACTCAG	AGCAGTCGCGAT	GT	CCTACGGGAGGCAGCAG

V3-341 F46	CCATCTCATCCCTGCGTGTCTCCGACTCAG	AGGACGCACTGT	GT	CCTACGGGAGGCAGCAG
V3-341 F47	CCATCTCATCCCTGCGTGTCTCCGACTCAG	AAGAGATGTCGA	GT	CCTACGGGAGGCAGCAG
V3-341 F48	CCATCTCATCCCTGCGTGTCTCCGACTCAG	ACAGCAGTGGTC	GT	CCTACGGGAGGCAGCAG
V3-341 F49	CCATCTCATCCCTGCGTGTCTCCGACTCAG	ACGTACTCAGTG	GT	CCTACGGGAGGCAGCAG
V3-341 F50	CCATCTCATCCCTGCGTGTCTCCGACTCAG	ACTCGCACAGGA	GT	CCTACGGGAGGCAGCAG
V3-518 R	CCTCTCTATGGGCAGTCGGTGAT	------------------	GT	ATTACCGCGGCTGCTGG

The V3-314F forward primer is complementary to positions 340-356, while the V3-518R reverse primer is complementary to positions 517-533 of the *Escherichia coli* 16S rDNA molecule *rrnB* GenBank accession number J01859.1. "------------------" indicates no sequence.

Table S2. Diagnostic primers for yeast detection.

Organisms	Gene	Forward Primer		Reverse Primer		Product size
Candida albicans	ITS1	CALB1	5'-ttt atc aac ttg tca cac cag a-3'	CALB2	5'-ttt atc aac ttg tca cac cag a-3'	273 pb
Nakaseomyses glabratus *	ITS2	CGL1	5'-tta tca cac gac tcg aca ct-3'	CGL2	5'-ccc aca tac tga tat ggc cta caa-3'	423 pb
Saccharomyces cerevisiae	MEX67	ScerF2	5'-gcg ctt tac att cag atc ccg ag-3'	ScerR2	5'-taa gtt ggt tgt cag caa gat tg-3'	150 bp

Pineda Díaz, Janet (Tesis (M.C.) Caracterización de la diversidad de la microbiota vaginal de mujeres mexicanas con candidiasis vulvovaginal recurrente Centro de Investigación y de Estudios Avanzados del I.P.N. Departamento de Genética y Biología Molecular, 2016). * former *Candida glabrata*.

Table S3. Sequencing summary.

Parameter	Control (n = 27)	Case (n = 54)
Total reads	2,012,838	3,758,769
Mean	74,549.56	69,606.83
sd	69,222.23	53,048.33
Median	56,828.00	55,813.50

n, number of samples

--end-of-text--
